# Soybean Meal-Dependent Intestinal Inflammation Induces Different Patterns of Bone-Loss in Adult Zebrafish Scale

**DOI:** 10.3390/biomedicines9040393

**Published:** 2021-04-06

**Authors:** Marta Carnovali, Roberto Valli, Giuseppe Banfi, Giovanni Porta, Massimo Mariotti

**Affiliations:** 1 IRCCS Istituto Ortopedico Galeazzi, 20161 Milan, Italy; marta.carnovali@grupposandonato.it (M.C.); banfi.giuseppe@hsr.it (G.B.); 2 Centro di Medicina Genomica, Department of Medicine and Surgery, University of Insubria, 21100 Varese, Italy; roberto.valli@uninsubria.it (R.V.); giovanni.porta@uninsubria.it (G.P.); 3 School of Medicine, Vita-Salute San Raffaele University, 20132 Milan, Italy; 4 Department of Biomedical, Surgical and Dental Sciences, University of Milan, 20122 Milan, Italy

**Keywords:** zebrafish, bone, osteoporosis, inflammatory bowel disease, intestinal inflammation, scale, soy

## Abstract

Inflammatory bowel disease have been linked to several health issues, including high risk of low bone mineral density. *Danio rerio* (zebrafish) is a good model to verify the effects of intestinal inflammation, since its gastrointestinal and immune systems are closely related to that of mammalians. Zebrafish is also a powerful model to study bone metabolism using the scale as the read-out model. Food strongly impacts zebrafish gut physiology, and it is well known that soybean meal induces intestinal inflammation. Adult zebrafish fed with defatted soybean meal (SBM) exhibited an intestinal inflammation evidenced by morphological alterations, inflammatory infiltrate, and increased mRNA expression of inflammatory cytokines (IL-1β, IL-6, IL-8, IL-10, TGFβ, TNF-α). The peak of acute intestinal inflammation, spanning between week 2 and 3, correlates with a transitory osteoporosis-like phenotype in the scale border. Later, a chronic inflammatory condition, associated with persistent IL-8 expression, correlates with the progression of resorption lacunae in the scale center. Both types of resorption lacunae were associated with intense osteoclastic tartrate-resistant acid phosphatase (TRAP) activity. After 3 weeks of SBM treatment, osteoclast activity decreased in the scale border but not in the center. At the same time, alkaline phosphatase (ALP) is activated in the border to repair the bone matrix. This model can contribute to elucidate in vivo the molecular mechanisms that links intestinal inflammation and bone metabolism in IBD.

## 1. Introduction

Inflammatory bowel disease (IBD) was linked to several health issues in human patients, including high risk of low bone mineral density and increased risk of fractures [1]. *Danio rerio* (zebrafish) is an excellent model to verify the various adverse effects of intestinal inflammation, including any effects on bone tissue. In fact, zebrafish is a powerful model to study bone tissue in the adult organism using the scale as read-out model due to its high accessibility, number of samples, bone tissue similarity, and transparency [2]. Furthermore, it must also be considered that the zebrafish immune system is highly related to that of mammals in both the innate and adaptive branches [3]. Moreover, the zebrafish gastrointestinal system is closely analogous to that of mammalians, even if it is simpler and lacks a definite stomach. In fact, the zebrafish intestinal tract consists of a twice-folded long tube located in the abdominal cavity and is arbitrarily divided based on morphological characteristics in the intestinal bulb, the mid and the posterior intestinal segments. Zebrafish intestine has comparable absorptive and secretory functions with those of mammals and is itself organized in concentric layers, even if supporting connective tissue layers are less complex and it lacks specific muscularis mucosa. Furthermore, zebrafish intestine is organized in irregular folds rather than well-defined crypts and real villi [4].

In the last decade, several gastrointestinal pathologies were reproduced in zebrafish using both chemicals and genetic models [5]. Chemical models were created using oxazolone, 2,4,6-trinitrobenzene sulfonic acid (TNBS), and dextran sodium sulfate (DSS) [5] and are characterized by increased expression of pro-inflammatory cytokines, such as interleukin 1 beta (IL-1β) and tumor necrosis factor alpha (TNFα); eosinophil infiltration [6]; and different degrees of morphological changes in the intestine and of impaired gut functions [5]. An elevated level of TNFα is considered a key factor to determine the establishment of IBD even in zebrafish since Marjoram et al. confirmed this correlation using an inflammation-responsive transgenic zebrafish line (TgBAC TNFα: GFP) [7].

Soybean meal (SBM) has become the primary protein source in fish diet replacing fishmeal thanks to its high availability and low cost. Nevertheless, studies reported that SBM induces enteritis, a type of intestinal inflammation, in aquaculture fish species, such as Atlantic salmon [8,9,10,11,12,13], rainbow trout [11], common carp [14], juvenile turbot [15], and zebrafish larvae [16] and that the actual inflammatory component of soy-based meal is saponin. Saponins are amphiphilic molecules, steroid or triterpenoid glycosides, common in a large number of plants and plant-derived products, with different biological activities [17]. Saponins’ mechanism of action in the intestinal membranes in vivo is not yet completely understood, but they certainly have the ability to increase the epithelial permeability of the intestine affecting nutrient uptake [18] and inducing intestinal inflammation in adult fish [19,20,21].

It is known that soluble factors like hormones, cytokines, growth factors are able to modulate bone metabolism. In particular, local and systemic inflammatory changes influence the bone microenvironment resulting in alterations leading to bone loss complications [22,23]. An understanding of the physiological and pathological mechanisms that regulates these changes, still largely unknown, will help to find new therapeutic strategies against bone-loss diseases associated to inflammatory conditions.

The aim of this study was to analyze bone tissue alterations in an adult zebrafish model due to experimental IBD induced by SBM treatment.

## 2. Materials and Methods

### 2.1. Ethic Statement

This experimentation was performed in the Zebrafish Laboratory (IRCCS R. Galeazzi, GSD Foundation, Milan, Italy) according to the Italian and European guidelines on research practice (EU Directive 2010/63/EU). Zebrafish experimentation and all protocols of this study were approved by Ministry of Health (authorization n. 742/2019-PR).

### 2.2. Animals

Adult 9 months old zebrafish of AB strain were maintained in the ZEBTEC© Bench Top system (Tecniplast Spa, Buguggiate, Italy) according to standard conditions [24]. During the experimentation fish were maintained at 28 °C in fish water from the aquarium system. In the experiment, 55 fish were used with three replications for a total of 165 fish. Five fish were used at the beginning of the test to check the physiological state of the population, then 25 fish were fed a standard diet and 25 an experimental diet. The experiment lasted 5 weeks, and every week, five fish from each group were sampled and analyzed.

### 2.3. Diets

Standard fish food (Vipagran, Sera GmbH, Heinsberg, Germany) was used as a control soy-free diet (CTR). The experimental diet was formulated based on a previous study performed on zebrafish larvae [16] that demonstrated that soy saponin, a protein found in low amount in soybean flour, is responsible for the intestinal inflammatory process. This diet ensured a high saponin content because we used soybean flour type I (Sigma Aldrich, St. Louis, Missouri, USA), a specifically defatted soybean flour with high protein content (protein ~52% (85+% dispersible and 1% fat)) and consequently an increased saponin concentration. The experimental diet (SBM) was composed of 50% *w/w* control diet and 50% *w/w* soybean flour, mixed with some water, then dried, and reduced in size to crumbs suitable for zebrafish consumption. Fish were fed with an automated system 3 times a day for 5 weeks (T1–T5).

### 2.4. Samples Collection

Fish were euthanized using a 300 mg/L tricaine methanesulfonate (Sigma Aldrich, St. Louis, MO, USA) solution [25]. Zebrafish intestines were isolated using sterile scalpels (Paragon^®^, Maersk Medical LTD, Sheffield, England) and stored in DNA/RNA Shield (Zymo Research, Irvine, CA, USA). Scales were carefully removed from either side of the fish body using Dumont^®^ Stainless Steel Forceps (Sigma Aldrich) and processed differently depending on the analysis to be performed as described below. All samples were isolated using a light stereomicroscope (Olympus SZX-ZB7, Olympus, Tokyo, Japan).

### 2.5. Histological Analysis and Morphometric Measures

Microscopic analyses were conducted in collaboration with Dr. Anna Maria Chiaravalli, U.O. di Istologia e Anatomia Patologica, Ospedale di Circolo, ASST-Sette Laghi, Viale L. Borri 57, 21,100 Varese, Italy. Fresh samples were fixed in a 10% formalin buffer for 12 h in a *w/w* 20:1 ratio. For perfect dehydration and fixing processes, we used the Donatello Fast (Diapath, Martinengo, Bergamo, Italy) automatic processor. Samples were then embedded in paraffin with the Leica EG1150H automated system. For optic microscopy, transversal sections of 8–10 µm thickness were obtained with the Leica biosystem RM2235 microtome (Leica, Wetzlar, Germany). Sections were then stained with hematoxylin-eosin and observed on an optic microscope (Leica DMRA, Laica, Wetzlar, Germany). Images were acquired with a Nikon D5-5M camera (Nikon, Tokyo, Japan), and the thickness of mucosa was evaluated using imaging software.

### 2.6. Myeloperoxidase Staining and Cell Count

Deparaffinization of 8 µm thick formalin fixed paraffin embedded (FFPE) sections was performed following standard protocols. Slides were mounted on poly-L-lysine-coated slides, heated in an oven (60 °C, 30 min) and rehydrated through an alcohol series to water. Endogenous activity was blocked with 3% hydrogen peroxide solution for 12 min and antigen retrieval was obtained by 10mM Citrate Buffer (pH 6) treatment. Slides were incubated overnight at 4 °C with a rabbit polyclonal anti-myeloperoxidase (MPO) primary antibody (ab210563, Abcam, Cambridge, England, UK; 1:100 dilution) followed by goat anti-rabbit IgG H&L (HRP) (ab6721, Abcam, Cambridge, England, UK; 1:200 dilution) secondary antibody for 1 h and ABC peroxidase complex. To localize MPO, 3,3′-diaminobenzidine tetrahydrochloride (DAB) was added. The immunohistochemical reaction was developed with diaminobenzidine–hydrogen peroxide reaction, and sections were counterstained with hematoxylin. The immune cells evaluation was performed by counting the MPO-positive cells present in a defined area of each histological section respect to total cells.

### 2.7. Intestinal Gene Expression Analysis and Inflammatory Status Estimation

Total RNA extraction was performed using the EuroGold Total RNA Mini kit (Euroclone, Milan, Italy) according to the manufacturer’s instructions, then RNA was quantified with Nanodrop^®^ ND 100 (Thermo Fisher Scientific, Waltham, MA, USA), and its integrity was verified on a 2% agarose gel. cDNA was synthesized from RNA samples with the High-Capacity cDNA synthesis kit (Applied Biosystems, Foster City, CA, USA) according to the manufacturer’s instructions. Quantitative real-time PCR (qRT-PCR) analysis was performed using AB PRISM 7000 (Applied Biosystem, Foster City, CA, USA) instrument with 30 ng of cDNA per well with the SYBR green (Power SYBR green Universal PCR Master Mix, Promega, Pero, Milan, Italy) method (thermal profile was: 50 °C for 2 min, 95 °C for 10 s and 40 cycles of 95 °C for 15 s, and 60°C for 1 min). Analyses were performed with the Ct method using β actin as an endogenous control. Then the average ΔCt value was calculated by subtracting the control ΔCt from the treated ΔCt, and the relative quantity of mRNA was calculated as 2-ΔΔCt [26]. The genes studied were *Interleukin-1β (IL-1β), Interleukin-6 (IL-6), Interleukin-8 (IL-8), Interleukin-10 (IL-10), Transforming growth factor-β (TGFβ)*, and *Tumor necrosis factor-α (TNF-α).* The PCR amplification was performed using the primers for *IL-1β* (sense: 5′-CAGATCCGCTTGCAATGA-3′; antisense: 5′-TTGTGCTGCGAAGTCCAC-3′), *IL-6* (sense: 5′-AAGGGGTCAGGATCAGCAC-3′; antisense: 5′-GCTGTAGATTCGCGTTAGACATC-3′), *IL-8* (sense: 5′-GCAAAATCATTTCAGTGTGTGTT-3′; antisense: 5′-CAGACCTCTCAAGCTCATTCC-3′), *IL-10* (sense: 5′-AACTCAAGCGGGATATGGTG-3′; antisense: 5′-GACCCCCTTTTCCTTCATCT-3′), *TGFβ* (sense: 5′-AATGGCTGCAGGGTTCAG-3′; antisense: 5′-GGTTTGCTTTACAGTCGCAGT-3′), and *TNFα* (sense: 5′-AGGCAATTTCACTTCCAAGG-3′; antisense: 5′-AGGTCTTTGATTCAGAGTTGTATCC-3′) designed with the Primer3 free online software (version 4.1.0, https://primer3.ut.ee/).

To estimate the actual intestinal inflammatory status, we slightly modified the inflammatory index formula created by Bond et al. [27]. Briefly, for each sampling time every detected level of the cytokines was converted to a “Z-score”. Then the “inflammatory index” was calculated as the sum of the Z-scores of *IL-1*, *IL-6*, *IL-8*, and *TNF-α*, while the “anti-inflammatory index” coincides with the *TGFβ* and *IL-10* Z-score. For each sampling time, the “anti-inflammatory index” was subtracted from the “inflammatory index” to obtain the “Total inflammatory index”.

### 2.8. Bone Matrix Vital Staining

To highlight the scale mineralized matrix resorption and its new deposition, fish were double-stained with successive pulses of vital staining according to the method published by Kimmel et al. [28]. At the beginning of the experiment, all fish were stained with 0.005% Alizarin Red S (Sigma Aldrich, St. Louis, MO, USA) solution overnight in the dark. Then fish were divided into the two diet groups, and at the end of every week of treatment, 5 SBM and 5 CTR fish were isolated and stained with 0.005% Calcein (Sigma-Aldrich) solution overnight in the dark. After repeated washes, scales were collected from these fish as previously described, fixed for 15 min in 3.5% formaldehyde 0.1 M sodium phosphate buffer solution, and analyzed using a fluorescence microscope (Olympus SZX-ZB7, Olympus, Tokyo, Japan) collecting and elaborating images with Discovery CH30 camera (Tiesselab, Milan, Italy).

### 2.9. Histological TRAP and ALP Assay in Scales

Histological tartrate resistant acid phosphatase (TRAP) activity was evaluated in the explanted scales using the Leukocytes Acid Phosphatase Detection Kit (Sigma-Aldrich) processing the samples according to manufacturer’s protocol. A histological ALP assay was performed using BCIP^®^/NBT liquid substrate (Sigma Aldrich) according to the manufacturer’s protocols. Histological assays, as well as biochemical assays, were performed separately after scale cut, since the zebrafish scale presents two different parts; the anterior part is composed mainly of bone tissue, and the posterior part is covered by epidermis. In this way it was possible to highlight the different bone marker expression in the two parts of the scale. Moreover, the epidermis has blood vessels that express high levels of ALP, compromising the efficiency of the histological ALP analysis.

### 2.10. Biochemical TRAP and ALP Scale Assays

Biochemical alkaline phosphatase (ALP) and TRAP activities were evaluated on explanted scales following previously published methods [29,30] and reading the absorbance levels at 405 nm using a spectrophotometer (iMarkTM Microplate Reader, Bio-Rad, Hercules, CA, USA).

### 2.11. Statistics

Five SBM fish and five CTR fish were tested at the end of each week of treatment, and the entire experiment was replicated three times. PCR experiments were repeated with comparable results at least three times and evaluated through statistical analysis using Student’s t-test. Significance values were set at *p* < 0.05 (*), *p* < 0.01 (**), and *p* < 0.001 (***). Biochemical scales analyses of ALP and TRAP activity were performed using 20 scales per fish for each analysis, while histological assays were performed on 50 scales. Bone matrix vital staining analysis was evaluated on 20 scales for each fish. All the collected data were analyzed using Student’s t-test and results expressed as mean ± standard deviation vs. control. Significance values were set at less than *p* < 0.05 (*), *p* < 0.01 (**), and *p* < 0.001 (***).

## 3. Results

### 3.1. Soybean Meal Induces Intestinal Inflammation in Adult Zebrafish

**Figure 1:**
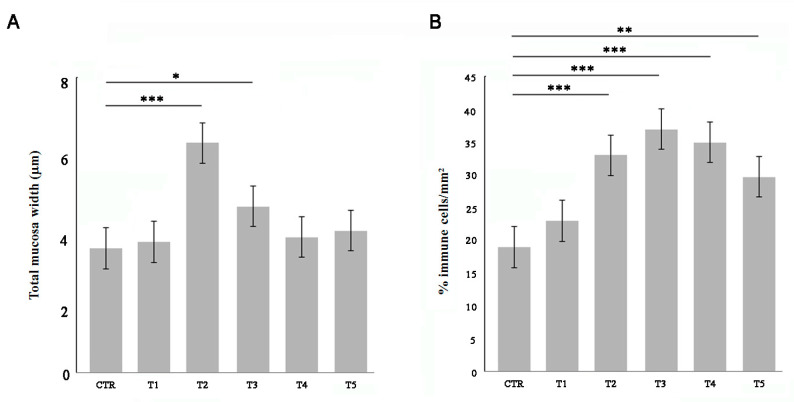
Morphometric and infiltrate analysis of the gut wall. Fish were fed with a soy-supplemented diet for one (T1), two (T2), three (T3), four (T4), and five (T5) weeks or normal diet (CTR). (**A**) The total wall thickness was found increased after two and three weeks of treatment (T2 vs. CTR, *p* < 0.001; T3 vs. CTR, *p* < 0.05). (**B**) The percentage of MPO-positive immune cells was found increased in the submucosal layer of SBM-treated fish starting from T2 (T2 vs. CTR, *p* < 0.001; T3 vs. CTR, *p* < 0.001; T4 vs. CTR, *p* < 0.001; T5 vs. CTR, *p* < 0.01). Significance values were set at less than *p* < 0.05 (*), *p* < 0.01 (**), and *p* < 0.001 (***).

Adult zebrafish were fed for 5 weeks with 50% *w/w* soybean meal (SBM) and compared with control soy-free diet (CTR). After each week of treatment, fish intestines were sampled for morphological and histochemical analysis of the gut wall. The total wall thickness significantly increased after two weeks of SBM treatment and then returned to the physiological state at T4 (Figure 1A). The percentage of immune cells which infiltrated the intestinal wall was evaluated by myeloperoxidase (MPO) staining. The SBM diet induced an increase of immune cells from two weeks in the intestinal mucosa (Figure 1B).

**Figure 2:**
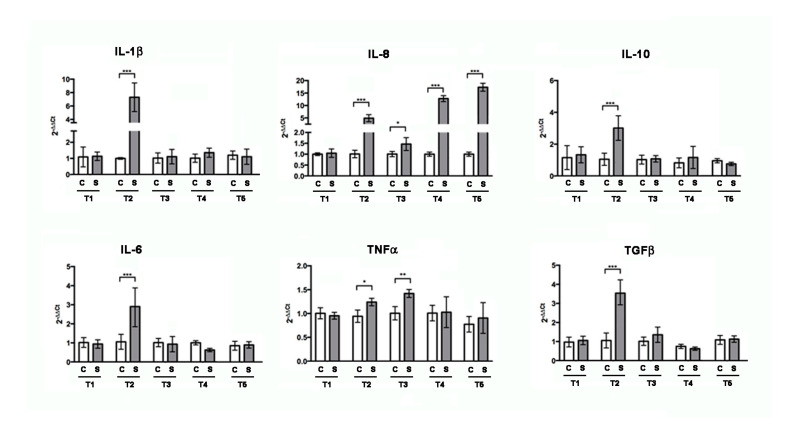
Gene expression analysis by RT-PCR for inflammatory marker genes on zebrafish intestine untreated (CTR) or treated with SBM for one (T1), two (T2), three (T3), four (T4), and five weeks (T5). IL-6, IL-10, TGFβ, IL-1β, IL-8, and TNFα resulted to be statistically significantly increased after two weeks (IL-1β: CTR vs. T2, *p* < 0.001; IL-8: CTR vs. T2, *p* < 0.001; IL-10: CTR vs. T2, *p* < 0.001; TGFβ: CTR vs. T2, *p* < 0.001; IL-6: CTR vs. T2, *p* < 0.001; TNFα: CTR vs. T2, *p* < 0.05) and IL-8 and TNFα after three weeks (IL-8: CTR vs. T3, *p* < 0.05; TNFα: CTR vs. T3, *p* < 0.01). After four and five weeks of treatment, only IL-8 resulted to be significantly increased (IL-8: CTR vs. T4, *p* < 0.001 and IL-8: CTR vs. T5, *p* < 0.001, respectively). Significance values were set at less than *p* < 0.05 (*), *p* < 0.01 (**), and *p* < 0.001 (***).

After 2 weeks of treatment, RT-PCR analysis (Figure 2) of intestinal total RNA revealed increased levels of pro-inflammatory cytokines IL-1β, IL-6, IL-8, and TNFα, as well as the anti-inflammatory cytokine TGFβ and IL-10. After 3 weeks of treatment, the cytokines gene expression returned to be comparable to control, except IL-8 and TNFα. Interestingly, after 4 and 5 weeks, only IL-8 increased again.

### 3.2. Soybean-Meal Induces a Transient Bone Resorption in the Scale Border

**Figure 3:**
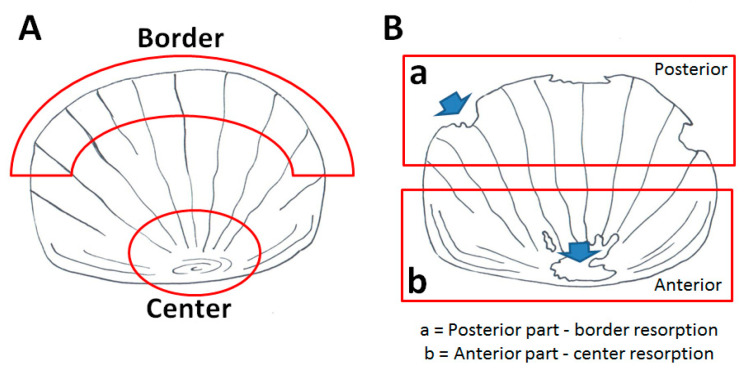
Schematic representation of the adult zebrafish scale (**A**) The “center” is located in the anterior part of the scale, whereas the “border” is in the posterior part. (**B**) Resorption lacunae in the posterior part are distributed along the border, while in the anterior part, they are concentrated in the center.

Bone alteration can be evaluated in the adult fish using the scale as a read-out system. Part of the dermal skeleton was anatomically characterized in Figure 3A.

**Figure 4:**
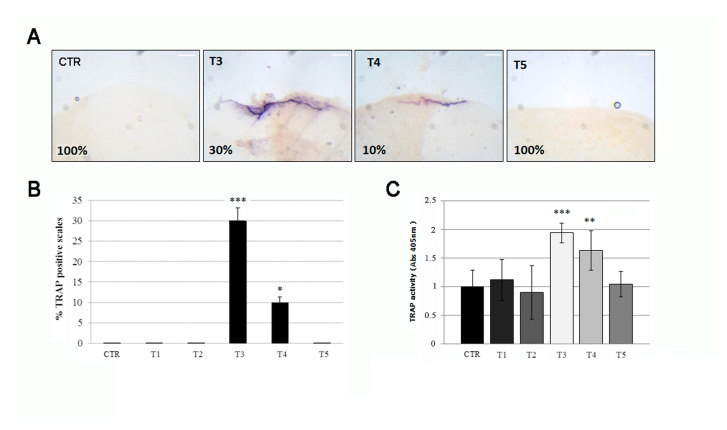
(**A**) The histological TRAP activity assay highlighted resorption activity along the anterior border of the scale after 3 (T3) and 4 (T4) weeks of treatment, whereas no activity was found in the scales of untreated fish (CTR), T1 (not shown), T2 (not shown), and T5. The percentage indicates the representation of the picture phenotype respect the total scales (scale bar: 0.1 mm). (**B**) The percentage of scales with detectable TRAP activity staining was 30% and 10%, respectively, at T3 and T4 (CTR vs. T3, *p* < 0.001; CTR vs. T4, *p* < 0.05). The value of the control at each experimental point was omitted, as it was identical to the initial CTR. (**C**) The biochemical TRAP activity assay indicated a peak in resorption activity in the scale after 3 weeks (T3) (CTR vs. T3, *p* < 0.001). Then it rapidly decreased at T4 (CTR vs. T4, *p* < 0.01) and returned to the control level at T5. The value of the control at each experimental point was omitted, as it was identical to the initial CTR. Significance values were set at less than *p* < 0.05 (*), *p* < 0.01 (**), and *p* < 0.001 (***).

Three weeks of SBM treatment induce the alteration of bone markers TRAP in fish scales, indicative of osteoclastic activity. The histological TRAP activity assay highlighted a relevant osteoclast activity along the scale borders of SBN-treated fish after 3 weeks (T3), whereas it became less intense at T4 and disappeared after 5 weeks (T5) (Figure 4A, black arrows). The percentage of scale borders with TRAP activity corresponds to 30% and 10%, respectively, at T3 and T4 (Figure 4B). Moreover, a significant TRAP activity can be detected in treated fish after 3 weeks (T3) by biochemical assay, followed by a rapid decrease at T4 and absence of signal at T5 (Figure 4C).

### 3.3. Anabolic Stimuli Rescued Osteoporotic Phenotype in the Scale Border

**Figure 5:**
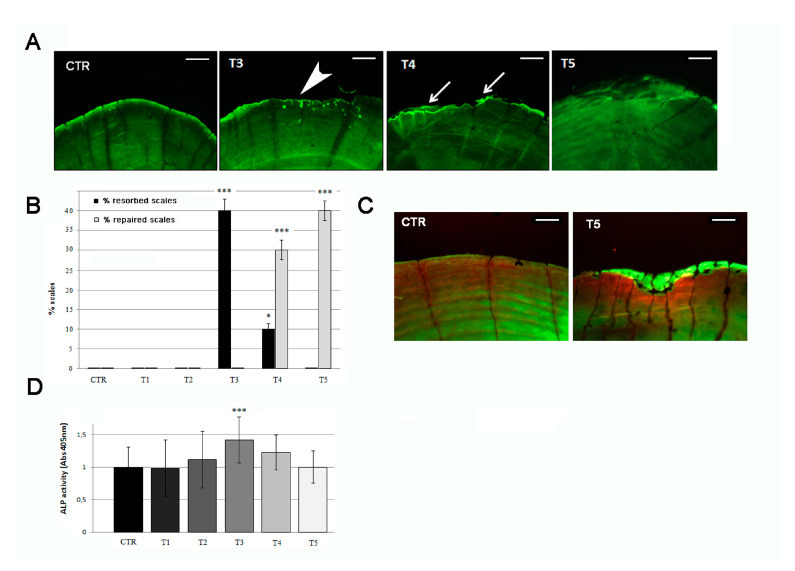
(**A**) Calcein vital staining of scales from treated fish and untreated controls. Control scale show normal profile, while scales from treated fish show bone resorption lacunae after 3 weeks of treatment (T3, white arrowhead). At 4 weeks of SBM treatment, the lacunae started to fill by new matrix deposition (T4, white arrows) and appeared completely repaired in the fifth week (T5) (scale bar: 0.1 mm). (**B**) Percentage of resorbed scales versus repaired scales. The percentage of resorbed scales decreased as the percentage or repaired scales increased (resorbed scales: CTR vs. T3, *p* < 0.001; CTR vs. T4, *p* < 0.05; repaired scales: CTR vs. T4, *p* < 0.001; CTR vs. T5, *p* < 0.001). (**C**) Alizarin Red-calcein double staining shows that the resorbed area was completely filled by new matrix at the end of the fifth week (green signal in T5) of SBM treatment in all scales. Untreated control (CTR) is shown as reference (scale bar: 0.07 mm). (**D**) Biochemical ALP activity assay indicates a peak in anabolic activity in the scale after 3 weeks of treatment (T3) (CTR vs. T3, *p* < 0.001). The value of the control at each experimental point was omitted as it was identical to the initial CTR. Significance values were set at less than *p* < 0.05 (*), and *p* < 0.001 (***).

Calcein live staining confirmed the presence of bone matrix resorption lacunae after 3 weeks of SBM treatment, detectable as bone-loss area in the posterior part of the scale, along the border (Figure 5A, T3, white arrowhead and Figure 3B) and covering an average of 4.5–5.0% of the scale surface (significant with *p* < 0.001). These resorption lacunae were filled by new mineralized matrix starting in the fourth week of treatment (Figure 5A, T4, white arrows) and concluded in the fifth week (Figure 5A, T5). New matrix deposition can be evidenced by more intense green staining filling the lacunae area. The percentage of resorbed scales reached 40% of the total in T3 but decreased in T4 (10%) and vanished at T5 (0%). In the meantime, the anabolic process, starting at T3, increased the percentage of repaired scales at T4 (30%) and terminated at T5 (40%) (Figure 5B). After 5 weeks, all lacunae were filled by new mineralized matrix in scale border (Figure 5C).

The analysis of the posterior part of the scales, highlights that SBM treatment does not reduce osteoblastic ALP activity. On the contrary, an up regulation was biochemically detected in treated fish after 3 weeks (T3), confirming the activation of bone anabolic functions in the border area (Figure 5D).

### 3.4. Soybean-Meal Induces a Permanent Bone Resorption in the Scale Center

**Figure 6:**
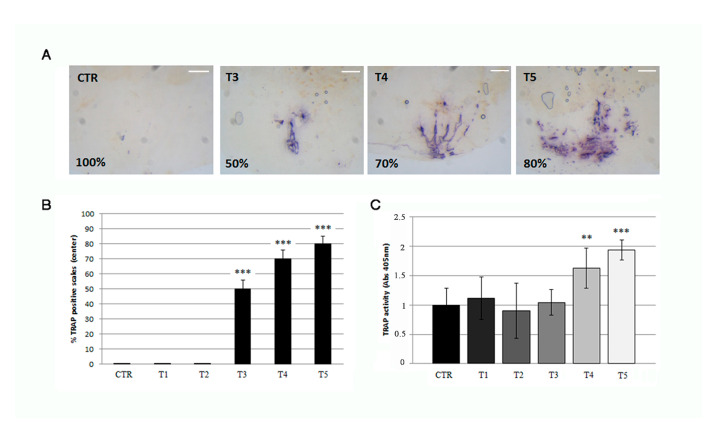
(**A**) Histological TRAP activity assay highlights resorption activity along the central area of the scale after 3 (T3), 4 (T4), and 5 (T5) weeks of treatment, whereas no activity is found in the scales of untreated fish (CTR), T1 (not shown) and T2 (not shown) (scale bar: 0.1 mm). (**B**) The percentage of scale with detectable TRAP activity staining is 50%, 70% and 80% respectively at T3, T4 and T5 (CTR vs. T3, *p* < 0.001; CTR vs. T4, *p* < 0.001 CTR vs. T5, *p* < 0.001). The value of the control at each experimental point was omitted as it was identical to the initial CTR. (**C**) Biochemical TRAP activity assay indicates a significant resorption activity in the center of scales after 4 and 5 weeks (T4 and T5) (CTR vs. T4, *p* < 0.01; CTR vs. T5, *p* < 0.001). The value of the control at each experimental point was omitted as it was identical to the initial CTR. Significance values were set at less than *p* < 0.01 (**), and *p* < 0.001 (***).

Histological TRAP activity assay detects another relevant osteoclast activity in the central area of the scale in 3 weeks (T3) SBM-treated fish, where the staining increased progressively from T3 to T5 as well as the resorbing surface (Figure 6A and Figure 3B). The percentage of scale with central TRAP activity corresponds to 50%, 70%, and 80%, respectively, at T3, T4 and T5 (Figure 6B). Moreover, a significant and progressive increase of TRAP activity was detected in SBM-fish after 3 weeks (T3) by biochemical assay (Figure 6C).

### 3.5. Osteoporotic Phenotype in the Scale Center Is Not Rescued by Anabolic Stimuli

**Figure 7:**
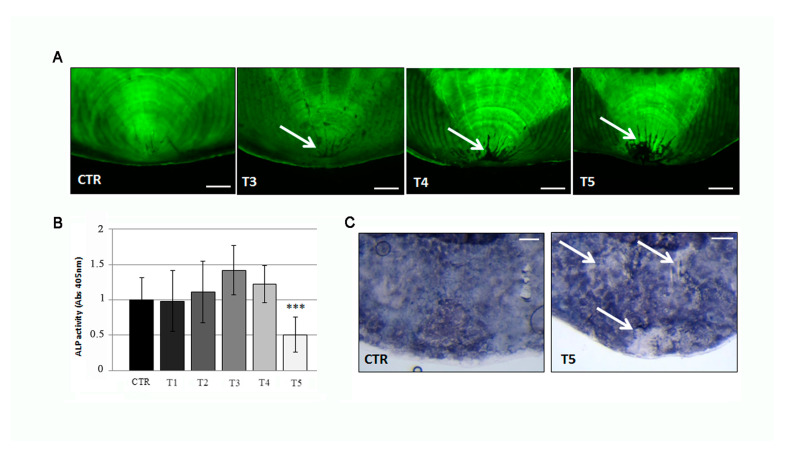
(**A**) Calcein vital staining of scales from treated fish and untreated controls. Control scale show normal profile, while scales from treated fish show central resorption lacunae after 3 weeks of treatment (T3, white arrow) (scale bar: 0.1 mm). (**B**) Biochemical ALP activity assay indicates a downregulation of anabolic activity in the scale after 5 weeks of treatment (T5) (CTR vs. T5, *p* < 0.001). The value of the control at each experimental point was omitted as it was identical to the initial CTR. (**C**) Histological ALP assay confirmed the loss of enzymatic activity (white arrows) in the center of scales after 5 weeks of SBM treatment (scale bar: 0.1 mm). Significance values were set at less than *p* < 0.001 (***).

Calcein live staining confirms the presence of resorption lacunae in 50%, 70% and 80% of scales after 3, 4, and 5 weeks, respectively, of SBM treatment and associated with a progressive increase of the resorbed area (Figure 7A, white arrowhead). Absence of new matrix deposition can be evidenced into the lacunae area. The SBM treatment reduced the biochemical ALP activity in the anterior half of the fish scales significantly at T5 (Figure 7B) which corresponds to a downregulation of ALP staining signal in the central area of SBM-fish scales (Figure 7C).

### 3.6. Different Inflammatory Stimuli Causes Different Bone Loss Patterns in Zebrafish Scales

**Figure 8:**
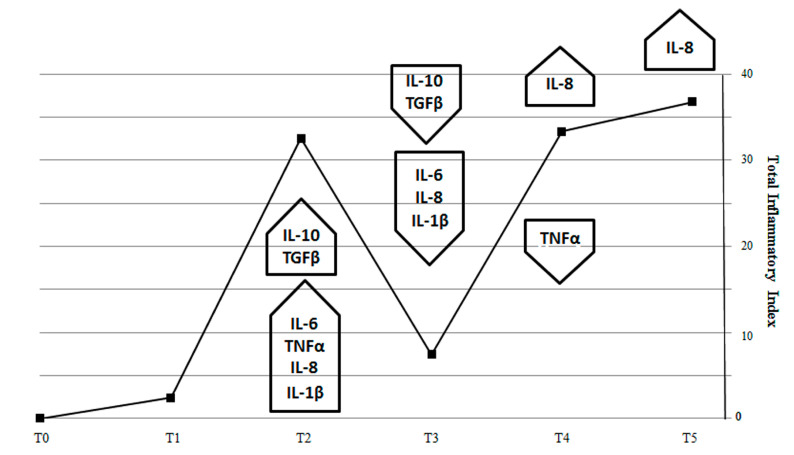
Total inflammatory index (TII) generated by expression of pro-inflammatory (*IL-1β*, *TNF-α*, *IL-8* and *IL-6)* and anti-inflammatory *(IL-10, TGFβ)* genes in fish intestine. Total inflammatory index peaks during the second week of SBM treatment to indicate the maximum activity of pro-inflammatory cytokines (IL-1β, TNF-α, IL-8, and IL-6). Next, TII dropped at T3, because of the effect of the anti-inflammatory cytokines TGFβ and IL-10 and returned to increase in T4 and T5 because of sustained IL-8 expression.

In order to correlate the course of intestinal inflammatory markers and the bone effects, we used the “inflammatory index” [27] to quantify the inflammation in our model. In this way, we can estimate that the “total inflammatory index” (TII) of SBM-treated fish generates a curve with maximal values at T2 and T4/5, which represent the two peaks of inflammatory activity in the intestine (Figure 8).

The effect of anti-inflammatory (IL-10 and TGFβ) cytokines counteracted the pro-inflammatory stimulus (IL-1β, IL-6, TNFα) in T2 and reduced the TII in T3, indicating that acute inflammation phenomenon was suppressed while a residual inflammatory stimulus remained at T3 with TNFα and IL-8 expression. Interestingly, TII reared up again when IL-8 returned to being expressed at high levels, suggesting that a chronic inflammation may substitute the acute one at fourth week.

**Figure 9:**
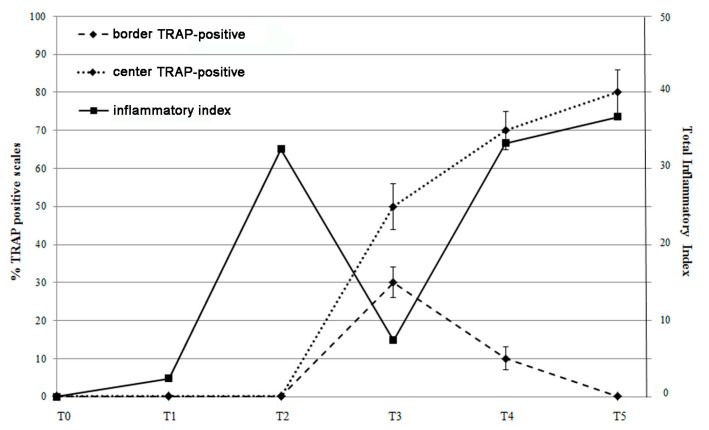
Correlation between the percentage of TRAP positive scales (center and border) and the total inflammatory index curve. TRAP activity in the scale border follows the inflammatory index peak at T2, while TRAP activity in the center follows the late inflammatory index curve (T4 and T5).

Comparing the inflammatory index trend with the percentage of TRAP positive scales (Figure 9), we observed that the resorbing activity in the scale border seems to be correlated to the inflammatory pick at T2 suggesting a bone response to an acute inflammatory stimulus. At T3, when the acute inflammatory state switched off, the resorbing activity was not supported anymore, and a boost of osteoblastic ALP activity stimulated osteoblast differentiation and activity in the refilling of border lacunae.

## 4. Discussion

In two weeks, adult zebrafish fed with SBM developed an acute intestinal inflammation associated with increase of mucosal thickness (edema), immune cell infiltrate, and expression of IL-1β and TNFα. These and other pro-inflammatory markers, IL-6 and IL-8, were widely raised after 3 weeks of SBM treatment to indicate a peak in an acute inflammatory phenomenon. These data are supported by a previous study performed by Hedrera et al. [16] where a strong increase in IL-1β and IL-8 mRNA levels was detected in zebrafish larvae fed with 50% SBM. IL-10, an anti-inflammatory cytokine that plays a role also in zebrafish intestine [5], was raised at T3 suggesting the activation of a compensatory anti-inflammatory response, usually documented in the acute form of inflammation. The increase of Il-10 completely turned off the intestinal inflammatory state in a week. The same behavior was demonstrated in adult common carp intestine, where a soy-based diet induced an intestinal acute inflammatory phenotype, from which fish recovered after three weeks, once the primary acute inflammatory stimulus was downregulated by IL-10 and TGFβ expression [14].

Pro-inflammatory and anti-inflammatory cytokines maintain a dynamic balance of the immune response in the health body, and this balance is dysregulated in IBD human patients [1]. The pro-inflammatory cytokines are well known to directly promote bone loss. IL-6 was identified as the main cytokine with a role in the pathogenesis of inflammatory-induced osteoporosis. Moreover it was reported that genetic variations of IL-6 and IL-1 receptor antagonist genes are strongly correlated with the clinical course of IBD and their bone complications [31].

Our data suggest a correspondence in fish between the intestinal inflammatory process and the osteoporosis in the scales. Interestingly, we also observed that two inflammatory peaks were temporally correlated with specific bone-loss phenotypes in the scales (Figure 3B). In summary, border lacunae are strictly associated with the acute inflammatory intestinal phenomenon evidenced at T3. The switch-off of an acute inflammatory peak allows the bone to repair the bone-loss along the borders. Similarly, in common carp intestine, a soy-based diet induced an acute inflammatory phenotype from which fish recovered after three weeks while TNFα remained upregulated until the fifth week [14]. Moreover, it was reported that an acute burst of bone loss represents an important adaptive program in acute inflammation where calcium and phosphorus are moved from bone to the non-skeletal tissues [32]. The bone loss that is seen in an acute flare of colitis appears to be very rapid at onset, but bone tissue can be successfully repaired in certain conditions [33].

On the other hand, central lacunae seem to be correlated to the late inflammatory peak (T4-T5). The engagement of IL-8 in a second inflammatory phase suggests that a low-grade pathological stimulus persists and stimulates a chronic inflammation state which supports a specific bone-loss pattern in the central part of the scale. In fact, IL-8 is able to induce osteoclastogenesis in different models [34,35,36].

It has been demonstrated that IL-8 can lead to chronic inflammatory conditions such as rheumatoid arthritis [37] and psoriasis [38]. In patients with IBD, both neutrophils and recruited macrophages are responsible for production of IL-8 [39], and recent human studies also demonstrated that early recurrent ileal lesions in Crohn’s patients were characterized by IL-8 production from neutrophils, whereas, in chronic lesions, IL-8 was produced by macrophages and T cells [40].

The presence of acute and chronic phases can be demonstrated in many inflammatory diseases associated with bone loss. For example, wear particles used of the artificial implant, produce an acute inflammatory reaction which becomes chronic, with progressive synovitis and bone destruction [41].

Different phases and localization of bone loss can be evidenced in many pathological situations; in patients with rheumatoid arthritis, early rapid loss of cortical diaphyseal bone is followed by gradual slowing [42]. Similarly, the glucocorticoid therapy causes first, a rapid decrease of the bone mineral density (BMD) of about 6% to 12% in the first year and an annual loss of about 3% thereafter [43].

On scale localization of bone resorption, further studies will elucidate the stimuli and the mechanisms driving the activation of osteoclasts in different areas of the scale.

The results highlight that zebrafish represent an excellent model to study IBD in adult organisms and in particular to study the inflammation-related complications. Zebrafish fed with SBM developed intestinal inflammation with different phases and different bone remodeling patterns. This model can contribute to elucidate in vivo the molecular mechanism and cellular cross-talk which regulate inflammation and bone remodeling.

## Data Availability

Not applicable.
